# Food system disruption: initial livelihood and dietary effects of COVID-19 on vegetable producers in India

**DOI:** 10.1007/s12571-020-01064-5

**Published:** 2020-07-14

**Authors:** Jody Harris, Lutz Depenbusch, Arshad Ahmad Pal, Ramakrishnan Madhavan Nair, Srinivasan Ramasamy

**Affiliations:** 1 World Vegetable Center, P.O. Box 1010, Bangkok, 10903 Thailand; 2 World Vegetable Center, South Asia, ICRISAT Campus, Patancheru, Hyderabad, Telangana 502324 India; 3 World Vegetable Center, Shanhua, Tainan, 74151 Taiwan

**Keywords:** India, Food systems, COVID-19, Diets, Vegetables, Livelihoods

## Abstract

Disruption to food systems and impacts on livelihoods and diets have been brought into sharp focus by the COVID-19 pandemic. We aimed to investigate effects of this multi-layered shock on production, sales, prices, incomes and diets for vegetable farmers in India as both producers and consumers of nutrient-dense foods. We undertook a rapid telephone survey with 448 farmers in 4 states, in one of the first studies to document the early impacts of the pandemic and policy responses on farming households.

We find that a majority of farmers report negative impacts on production, sales, prices and incomes. Over 80% of farms reported some decline in sales, and over 20% of farms reported devastating declines (sold almost nothing). Price reductions were reported by over 80% of farmers, and reductions by more than half for 50% of farmers. Similarly, farm income reportedly dropped for 90% of farms, and by more than half for 60%.

Of surveyed households, 62% reported disruptions to their diets. A majority of farm households reported reduced ability to access the most nutrient-dense foods. Around 80% of households reported ability to protect their staple food consumption, and the largest falls in consumption were in fruit and animal source foods other than dairy, in around half of households. Reported vegetable consumption fell in almost 30% of households, but vegetables were also the only food group where consumption increased for some, in around 15% of households.

Our data suggest higher vulnerability of female farmers in terms of both livelihoods and diet, and differential effects on smaller and larger farms, meaning different farms may require different types of support in order to continue to function. Farms reported diverse coping strategies to maintain sales, though often with negative implications for reported incomes. The ability to consume one’s own produce may be somewhat protective of diets when other routes to food access fail.

The impacts of COVID-19 and subsequent policy responses on both livelihoods and diets in horticultural households risk rolling back the impressive economic and nutrition gains India has seen over the past decade. Food systems, and particularly those making available the most nutrient-dense foods, must be considered in ongoing and future government responses.

## Introduction

Disruption to food systems has been brought into sharp focus by the COVID-19 pandemic, but such disruptions are a persistent feature of these complex systems, so it is important that we learn from the current crisis to be better prepared for the next one. This paper uses novel empirical data to understand disruptions to production, livelihoods and diets in agricultural households in India, to draw lessons from COVID-19 – and particularly its effects on nutrient-dense perishable food items – for making food systems more resilient. The objective is to gain a better understanding of how food system disruption affects the various roles of vegetables in food systems, and we draw on food systems theory and frameworks, and research on food system effects of previous shocks, to frame our study in the Indian context.

Food systems have been defined as “all the elements [...] and activities that relate to the production, processing, distribution, preparation and consumption of food, and the output of these activities” (HLPE 2014). Key outputs are the diets that people can access through the food system; the livelihoods of those involved across the food system; and the environmental effects of the food system. These complex and spontaneous systems are shaped by a range of drivers, from the biophysical and technological to the political and socio-cultural (HLPE 2020). When there are disruptions to these drivers, there is potential disruption to food systems.

Importantly, different foods are likely to be affected differently by food system shocks. Four months into the COVID-19 pandemic, the UN Food and Agriculture Organisation (FAO 2020) stresses that global stocks of staple food crops such as rice and wheat are adequate and 2020 looks set for a good harvest, depending on how long the pandemic and restrictions last. Perishable foods however – such as dairy, meat, fruit and vegetables – cannot be easily transported and stored and are more vulnerable to food system disruption. Restrictions to movement – whether of people, trucks or ships – have significant potential to disrupt production and trade in vegetables and their inputs, and their perishability means this may result in vastly increased food waste at a time when production is uncertain. It is precisely these perishable foods that are the most nutritious (having the most nutrients per calorie) (Beal et al. 2017) so concerns about food system disruptions also play out in concerns over reductions in diet quality.

Some of these hypothesised impacts from food systems theory have been studied in previous shocks, and signal some areas of the food system that will be important to understand in the current crisis. In terms of production, a study using a system dynamics model suggested that a severe pandemic with >25% reduction in labour availability can create widespread food shortages even in developed nations (Huff et al. 2015). This prediction was in fact witnessed during the Ebola epidemic in West Africa that began in 2014, where movement and labour were severely curtained and production volume of staple crops was reduced by 12% (Huber et al. 2018). In contexts where shocks lead to food gluts or shortages, a second key area is effects on food prices. Often the most nutritious foods increase most: In Indonesia a drought and financial shock in 1997–8 led to a 200% increase in the price of leafy greens alongside smaller rises in other food prices, for instance (Block et al. 2004). A 50% rise in staple food prices during the 2008 food price crisis led to a 21% increase in total food expenditure across low- and middle-income countries, putting pressure on household food baskets (Darnton-Hill and Cogill 2010).

Healthy diets based on diverse plant foods are already too expensive for over 1.5 billion people in the world (Hirvonen et al. 2020), and shocks such as COVID-19 that reduce incomes or increase prices will only exacerbate this situation. Reductions in diet quality (even while maintaining sufficient calories) has been seen in other significant food system shocks, with households tending to protect staple food consumption over the consumption of more expensive but more nutrient-dense foods (Darnton-Hill and Cogill 2010). In the Indonesia example, consumption of eggs fell by over half, and of green leafy vegetables by up to 30% (Block et al. 2004), severely limiting diet quality. Different populations are also affected differently: While COVID-19 does not distinguish between rich and poor, the diets of the most marginalized in society will be most affected and the least able to adapt. The coping strategies employed by farming households during shocks (such as borrowing and relying on social networks) are likely to have broader welfare implications in the longer term that need to be captured and understood (Galiano and Vera-Hernández 2008).

Overall, the type of shock matters: A biophysical shock affects food production and availability, and thereafter perhaps prices (Béné et al. 2015). An economic shock affects farmers’ ability to procure inputs and labour, but also consumers’ ability to afford food and therefore demand (Block et al. 2004). A health shock experienced directly by a household leads to changes in health expenditures and reductions in labour, either in the short term or longer term depending on the type of pandemic (HIV being chronic, Ebola being acute, for instance) (Harris 2014; Gillespie 2008). The COVID-19 pandemic (an acute health shock) and its associated social and policy responses (broader production and economic shocks) are exceptional in that they potentially affect multiple food system drivers at the same time; affect the food system from inputs and production to trade and marketing to price and affordability to consumer demand; and affect almost every scale, from local to global.

In this crisis, it is important to understand how farmers experience the shock of COVID-19 as both producers and consumers of food. The produce of vegetable farmers in particular is likely to be among the most disrupted, as outlined above. Our hypothesis based on previous research is that production might be disrupted and sales might fall for these farmers, impacting livelihoods; reduced incomes are likely to lead to less diverse and healthy diets in these households; and these impacts will be different for different types of farmers across socio-economic groups. We therefore aimed to understand impacts on the livelihoods and diets of vegetable farmers in India through the multi-layered shock of COVID-19.

## Methods and data

### Context

India reported its first case of COVID-19 on January 30th 2020, and early response measures related to the limiting of international travel and health system preparedness. It was only with increasing cases in late March that individual states ordered lockdowns, and on March 24th 2020 a full national lockdown was ordered, extended several times and still in place at the time of writing, though with relaxations applied according to the severity of COVID-19 in different zones. The lockdown coincided with peak harvest (*Rabi*) season for certain fruits and vegetables in many parts of India (FAO 2020), and agricultural work was largely allowed to continue, with agricultural operations remaining out of the purview of the COVID-19 lockdown restrictions (Padhee and Pingali 2020) except in active containment zone areas. Public and private transport restrictions however limited the domestic movement of seasonal workers and agricultural inputs, especially crop protection products, and significant negative impact was realized in terms of accessing markets for sales. Sudden closure of outlets left vegetable producers with reduced customer base, with street food vendors, restaurants and supermarkets mandated to close, though small food shops and open-air markets were allowed to open with time restrictions (GAIN 2020).

The current study was undertaken with farmers engaged in existing, forthcoming, and recently concluded projects with the World Vegetable Center (WorldVeg) in four Indian states (Table 1). Data were collected between May 5th–12th 2020, six weeks into the national lockdown and in the early stages of various government relief packages. In Jharkhand, about 10% of the vegetable produce was procured by the project itself during the lockdown; about 14% of the farmers we interviewed in Jharkhand were part of this initiative, and the JOHAR project was explicitly working with women farmers. In Andhra Pradesh, about 10% of the tomatoes produced were supplied to a processing factory through the project and its partners; about 50% of the farmers we interviewed in Andhra Pradesh were part of this initiative.

**Table 1:** Overview of project contexts

State	Project acronym	Aims	Vegetables
Jharkhand	JOHAR	Enhancing productivity through on farm demonstration and skills building (mainly women)	Multiple^1^
	CInI	Empowering and supporting farmers through better communities of practice	Multiple^1^
Assam	APART	Adding value and improving resilience of selected agriculture value chains	Multiple^2^
Andhra Pradesh	GIC	Enhancing the production, productivity and profitability of value chains	Tomato
Karnataka	Bhoo Samruddhi	Improving the farm productivity and livelihood of smallholder farmers	Multiple^3^
	RKVY	Increasing the income of farmers through effective technological interventions across the value chain	Onion

Notes: Project acronyms: Jharkhand Opportunities for Harnessing Rural Growth (JOHAR); Improved Livelihoods through Crop Diversification into Vegetables in Jharkhand and Odisha (CInI); Assam Agribusiness & Rural Transformation Project (APART); Green Innovation Centre (GIC); Rashtriya Krishi Vikas Yojana (RKVY)

^1^Tomato, Eggplant, Chili, Cabbage, Cauliflower, French bean, Green peas, Carrot, Okra, Cucumber, Bitter gourd, Bottle gourd and Watermelon

^2^Eggplant, Cabbage, Cauliflower, Tomato, Pumpkin, Black gram, Lentil and Pea

^3^Tomato, Chili, Capsicum, Cluster bean, Onion

## Study approach

The study used a phone-based survey method to elicit information about how vegetable farmers in four Indian states were affected by the lockdown measures imposed to contain COVID-19. Phone-based interviews were necessary because it was not possible to visit farmers in person. WorldVeg staff identified farmers from their projects with whom they worked directly and for whom they had phone numbers, and purposively selected farmers to call until they reached their sample size quota of 30 farmers. As only one single staff member was available in the projects in Andhra Pradesh and Karnataka, their quota was raised to 50. The quota followed a rule-of-thumb as we did not have information available for the appropriate power calculations. Responses were recorded by the staff members in real time using a customized smartphone application which reported and aggregated the data. Informed consent was sought and recorded before each interview. The study was virtually risk free for participants and the study plan was therefore exempted from ethical review by the Institutional Biosafety and Research Ethics Committee of WorldVeg before implementation.

We aimed to limit the interviews to 15 min and therefore included only 25 questions in the survey. These covered questions on production and wastage (vegetables produced, changes in production, reasons, and mitigation strategies); questions on prices, sales, and farm income (changes in each, reasons, and mitigation strategies); and questions on diets (changes in consumption of different food groups, reasons, and mitigation strategies). Change in diets was asked as a binary question (‘Has your household diet changed as a result of COVID-19’) and we also looked at changes in the balance of the individual food groups consumed. Importantly, each of the questions relied on farmers’ perceptions of change since COVID-19; we did not define a timescale but rather let the respondents attribute change to COVID-19 as they viewed it. Change within each of the quantitative questions was captured as a Likert scale (For instance: Increased a lot (more than doubled)|Increased a little (less than doubled)|No change| Decreased a little (not to half)|Decreased a lot (less than half)) sometimes presented with a percentage range (for instance: Sold nothing at all (>90%)|Lost most of sales (60%–90%)|Lost half of sales (40%–60%)|Lost some sales (10%–40%)|Lost little to no sales (<10%)|Increased sales). Qualitative questions (such as reasons and coping strategies) were asked without pre-defined responses, with enumerators instructed to probe for multiple answers and select the most appropriate responses from a multiple-choice list. We also collected some basic farm and household data in order to compare farms and households on proxies for key socio-economic issues (gender of the farmer, as a proxy for gender differences; and farm size, as a proxy for income/economic status).

Of the initial sample of 502 farmers contacted for this study, 40 rejected the interview, 11 had not produced vegetables, and 3 provided incomplete information, therefore the analytical sample had observations for 448 farmers. Descriptive analyses were undertaken for each topic, with particular sections also analysed by gender and farm size (larger or smaller than 1 ha (ha)) to understand heterogeneous effects. Ordered logit models were used to estimate the influence of farm and household characteristics on selected outcomes.

We use ordered logit regressions to analyse associations between the intensity of self-reported changes in vegetable livelihoods (quantity sold, prices, income) and diets (change in consumption per food group) due to COVID-19 and major household characteristics (farm size, gender of the farmer, and the number of produced vegetables). The dependent variables are on 5 to 6-point Likert scales. Where necessary we invert the order of the variables, so that higher values always reflect a worse situation of the household. To control for differences between the projects and their context, we add a set of state dummies to the list of independent variables.

## Results

### Farm and household characteristics

Across our survey a quarter of farmers were women (Table 2). This was driven by the project in Jharkhand where half of the respondents were women, while in all other states very few farmers were women (0–6%). Jharkhand also had much smaller farm sizes (average 0.8 ha) compared to the 1.5 to 3 ha farms in the other three states. In disaggregated analyses (not shown here) female farmers had slightly smaller farms on average than their male counterparts. Overall, 87% of farmers reported disruptions to production; 79% to sales; and 62% to diets.

**Table 2:** Farm and household characteristics, and overall COVID-19 effects

		Andhra Pradesh	Assam	Jharkhand	Karnataka	Total
Observations	n	29	163	200	56	448
Female farmer	%	3	6	51	0	25
Farm size	ha	2.89	1.48	0.80	2.77	1.43
	SD	(5.15)	(1.02)	(0.81)	(1.12)	(1.74)
*Self-reported impacts of COVID-19 (binary):*						
–Veg. production disrupted	%	76	77	94	100	87
–Unable to sell part of veg. Harvest	%	83	75	76	100	79
–Diet changed	%	21	46	90	30	62

Note: ha = hectares; SD = standard deviation

**Fig. 1:**
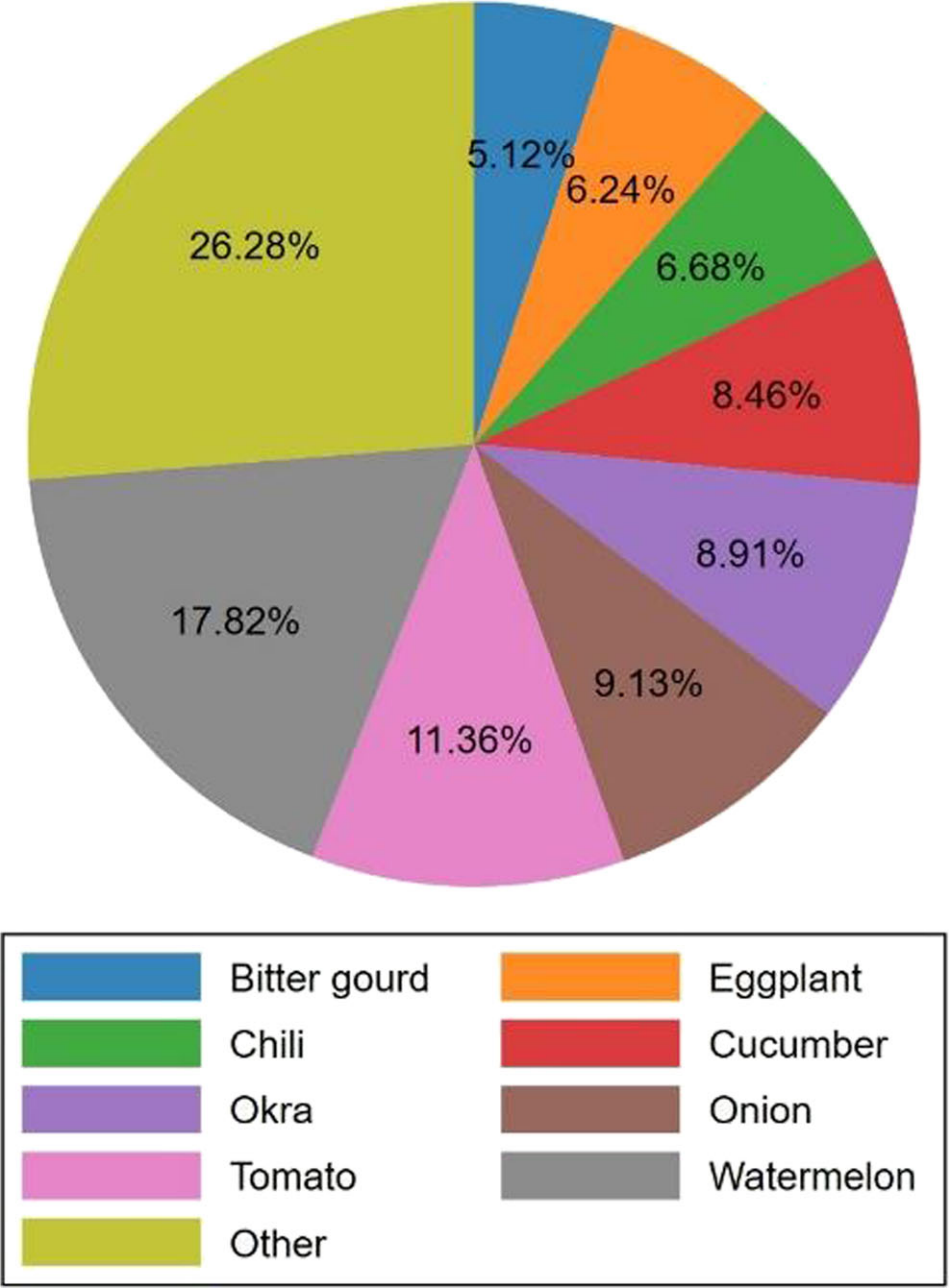
Major vegetables produced in the study areas during the survey

Farmers produced a diverse range of vegetables. The median number of vegetables produced was 3, the mean 4, and 30% of households produced five or more types of vegetable. This varied by state, with farmers in Andhra Pradesh mainly producing tomatoes, but farmers in other states producing 4–5 vegetables on average. Figure 1 shows the main vegetables produced by each farmer (self-reported), to which questions on production, sales and prices related. The ‘other’ vegetable category included several cucurbit species, beans, radish, beetroot and cauliflower, each of which was produced by less than 5% of farmers and combined into one collective category for visualization.

## Production, sales and prices

Of all types and locations of farms with interruptions to their production, most reported that prices were too low to continue with production (69%) or that they could not find buyers (65%). Of problems associated with production, most cited lack of transport (61%), many cited lack of inputs (39%), lack of harvest labour (32%), and some cited lack of storage (12%). Again this varied by state, though transport was cited in every state. Women farmers were less likely to suffer from lack of labour (but had smaller farms on average) but were more likely to see prices as too low. Large farms were more likely to lack labour and storage, and smaller farms had difficulty accessing inputs, in particular.

Farmers who experienced reduction in sales due to COVID-19 sold their produce through multiple marketing channels, from selling directly to consumers through fresh markets, to selling through collectors/middlemen (Table 3). Only two farmers sold directly to institutions, and another two to exporters or supermarkets. More female farmers sold directly to consumers and did not have supply contracts, and more male farmers sold through collectors and had a higher diversity of sales channels. The average number of marketing channels among smaller farms (<1 ha) was 0.6 less than among larger farms.

**Table 3:** Major marketing channels by gender

		All farmers	Male farmers	Female farmers	Diff. (Male – Female)	Std. Err.
Observations	n	353	269	84		
Sold directly to consumer	%	46	42	60	−17.89***	6.14
Sold directly to hotel, restaurant, school, or other institution		1	1	0	0.74	0.52
Picked up by local collector at farm gate		53	59	33	25.40***	5.96
Delivered to local collector		65	69	51	17.58***	6.14
Picked up by collector from other district, farm gate		15	17	7	9.96**	3.63
Delivered to collector from other district		14	14	13	1.03	4.25
Picked up by supermarket or exporter at farm gate		0	0	0	0.37	0.37
Delivered to supermarket or exporter		0	0	0	0.37	0.37
Collective marketing		3	1	7	−5.66***	2.91
Number of supply channels used	n	1.96	2.03	1.71	0.32***	0.12
Contract w. at least one supply channel	%	28	31	18	13.00***	5.04

Note: The number of supply channels is tested for equality of means (t-test), all others comparison test for equality of proportions (z-test), sample are all households that have been unable to sell some of their vegetables due to COVID-19, *N* = 353 ^*^ *p* < 0.1, ^**^ *p* < 0.05, ^***^ *p* < 0.01

**Fig. 2:**
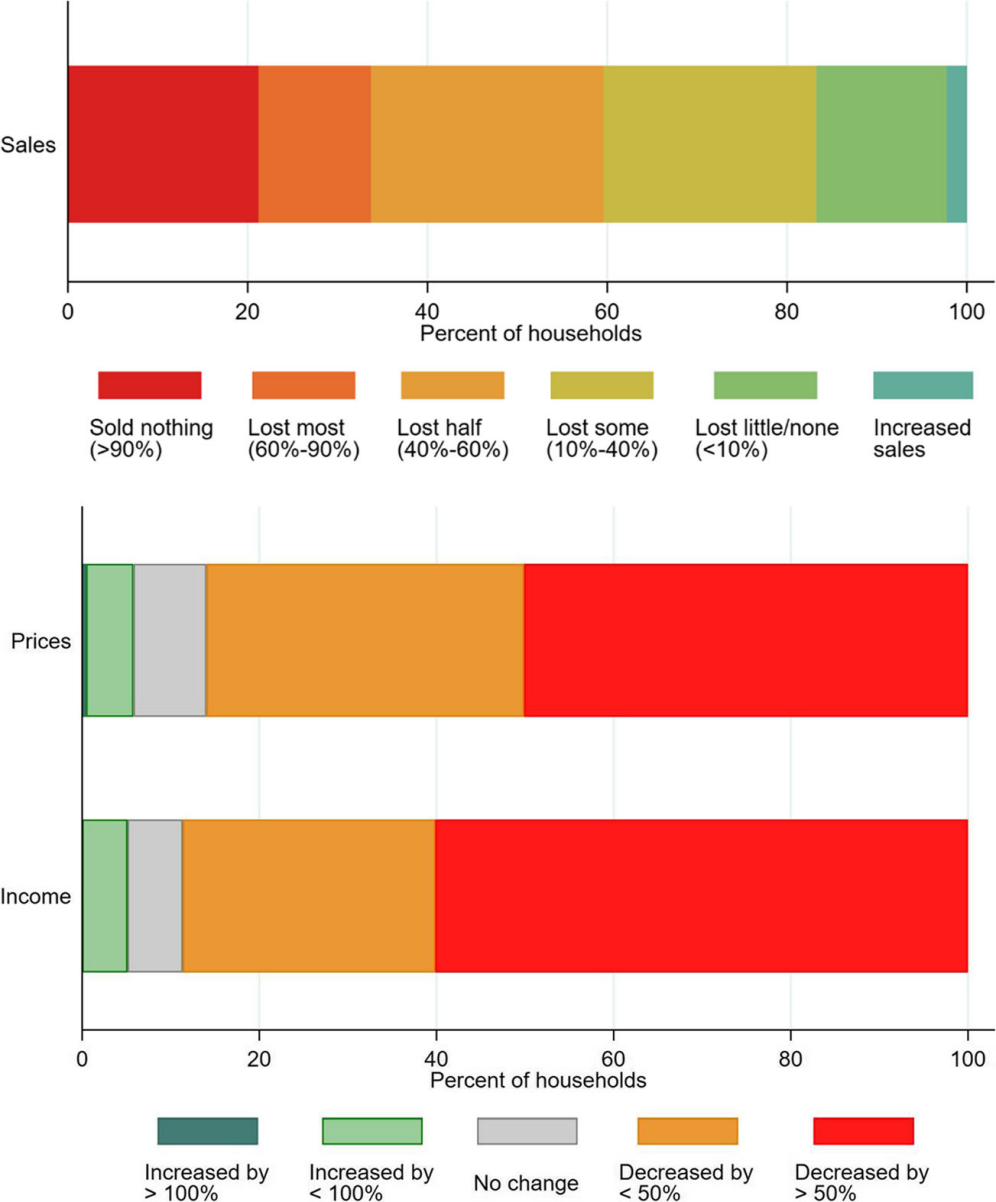
Reported changes in sales, prices and income

A vast majority of farmers saw declines in vegetable sales, prices and farm incomes (Fig. 2). Over 80% of farms saw some decline in sales, and over 20% of farms saw devastating losses of sales (sold almost nothing), slightly higher among female farmers, and double among small farms compared to large (data not shown here). Prices reduced for over 80% of farmers, and by more than half for 50% of farmers. Similarly, farm income dropped by for 90% of farms, and by more than half for 60%.

We estimated an ordered logit model to assess the association of farm size, gender of the farmer, and the number of produced vegetables with COVID-19 related shocks (Table 4). The results show that a higher diversity of produced vegetables is significantly associated with a better development of sales but worse changes in prices and income. Women experienced a stronger disruption to the price of their vegetables. Farm size was not significantly associated with any of the variables.

**Table 4:** Likelihood of experiencing worse effect on vegetable farming as a result of COVID-19, odds ratios

	Change in veg. Sales	Change in veg. Prices	Change in farming income
Observations	448	448	448
Farm size, ha	0.99	0.97	1.04
	(0.02)	(0.06)	(0.07)
Female farmer	0.80	1.64**	1.46
	(0.22)	(0.39)	(0.35)
Number of produced vegetable species	0.85***	1.21***	1.17***
	(0.03)	(0.05)	(0.05)
State dummies	X	X	X

Note: Ordered logit regressions. The categories of the independent variables are identical with those in Fig. 2 but in reversed order. Coefficients are displayed as odds ratios. An odds ratio of 1.00 indicates the absence of an association with the dependent variable, larger values indicate an association with a larger damage from COVID-19, and smaller values indicate an association with smaller damages or improvements. Results for state dummies are not displayed. Robust standard errors in parentheses, ^*^ *p* < 0.1, ^**^ *p* < 0.05, ^***^ *p* < 0.01

## Diets

**Fig. 3:**
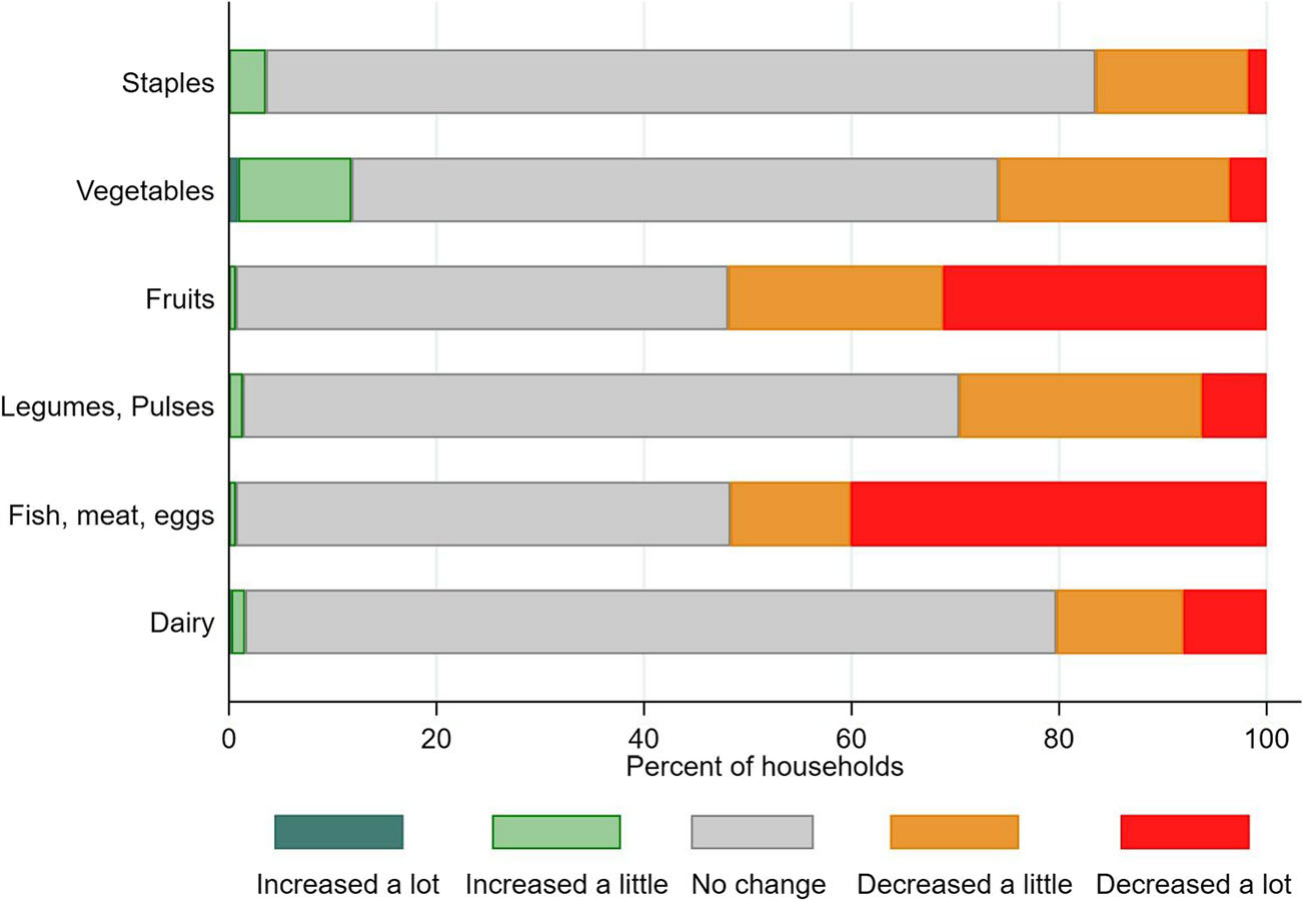
Self-reported change in diets among vegetable producing households in India since COVID-19

Of surveyed households, 62% reported disruptions to their diets. A majority of farm households reported ability to protect their staple food consumption, meaning their food security in terms of calories (Fig. 3), though 17% of households did report a fall in ability to procure staple foods. The largest falls in consumption were in fruit and animal source foods other than dairy, in around half of households. Pulse, dairy and vegetable consumption fell in 20–30% of households. Vegetables were the only food group where consumption reportedly increased in a significant proportion of households, with around 12% reporting an increase. The major reason for increases in vegetable intakes was eating the farm’s own production, while reasons reported for reductions in vegetable consumption were mostly due to reduced physical availability and affordability. Women farmers were significantly more likely than men to report a stronger reduction in consumption of vegetables, fruits, and dairy, controlling for other factors (*p* < 0.1 in separate ordered logit regressions), and were much less likely to be able to afford vegetables than men (42% of women versus 15% of men specifically quoting price changes; 38% of women versus 20% of men quoting changes in general affordability; data not shown here).

## Coping, mitigation and resilience

We asked about coping strategies used by farm households to mitigate the impacts of COVID-19 on sales, income and diets. In terms of farm income (Table 5), the most common strategies have been to find new markets (including selling door-to-door), reduce prices, and eat the farm’s own production. All of these are practiced more by female than male farmers. Women were only half as likely as men to apply no coping strategy for production. To deal with reduction in sales, most were leaving harvest in the field, feeding vegetables to livestock, and sharing vegetables with others. Smaller farms were generally less likely to reduce their production or destroy the harvest (plant less, leave vegetables in the field, compost vegetables, or feed to livestock). To mitigate the effects on farm incomes, most are finding new markets and reducing prices, with female farmers reducing prices more than men, and small farms more than large farms. In total 16% of farmers plan to produce less, while this rate differs between 57% in Karnataka and just 7% in Assam (data not shown here).

**Table 5:** Strategies of male and female farmers to cope with the effect of COVID-19 on farm income

		All farmers	Male farmers	Female farmers	Diff.(Male – Female)	Std. Err.
Observations	n	448	336	112		
No mitigation strategy	%	21	24	12	12.80***	3.83
Produce less		17	18	11	7.74*	3.61
Store more		5	6	1	5.36**	1.59
Process more		2	2	1	0.89	1.15
Find new markets		52	46	72	−26.79***	5.03
Reduce price		25	18	48	−30.36***	5.16
Eat own production		18	16	24	−8.04*	4.51
Adapt crop choice		2	3	1	2.08	1.28

Note: Test for equality of proportions of the two group applying each strategy (z-test), respondents were able to quote more than one coping mechanism ^*^ *p* < 0.1, ^**^ *p* < 0.05, ^***^ *p* < 0.01

In terms of dietary coping strategies, the most common were reducing household expenses and eating more own-produced food (Table 6). Other households relied on financial coping strategies, such as buying cheaper food, and borrowing money. Most coping strategies were used more by households in which the farmer is female, particularly the financial strategies; and these households were almost twice as likely to have received food aid or other formal support.

**Table 6:** Strategies of male and female farmers to cope with the effect of COVID-19 on diets

		All farmers	Male farmers	Female farmers	Diff. (Male – Female)	Std. Err.
Observations	n	448	336	112		
None/Not applicable	%	18	20	11	9.23**	3.65
Reduce household expenses		51	43	73	−30.06***	4.98
Eat more own-produced food		49	48	53	−5.06	5.45
Eat less		8	5	16	−11.01***	3.67
Buy cheaper food		12	6	30	−24.11***	4.54
Borrow money		14	10	28	−18.15***	4.52
Find other work		16	20	4	15.18***	2.92
Share with community		18	21	9	11.61***	3.48
Received food aid or other formal support		40	33	61	−27.98***	5.28

Note: Test for equality of proportions of the two group applying each strategy (z-test), respondents were able to quote more than one coping mechanism,^*^ *p* < 0.1, ^**^ *p* < 0.05, ^***^ *p* < 0.01

## Discussion

This study has shown food system effects of COVID-19 reported by farmers on production, livelihoods, food environments and diets in households involved in vegetable production in four Indian states. Our data is limited by its convenience sample and moderate sample sizes across different states, limiting the generalizability of the findings. As with all surveys, the findings rely on the accuracy of the data portrayed by the respondents, and we asked specifically about the perceptions of farmers on changes since COVID-19 rather than use independently verifiable data. Our focus on vegetable farmers was deliberate, because we wanted to understand the impacts on a sector providing nutritious foods, but of course our findings cannot necessarily be generalized to farmers of other crops, or to households relying on other livelihoods. Nonetheless, this is one of the first studies to document the early impacts of the pandemic and policy responses on farming households, and provides an important snapshot of impacts on a sector that provides both decent livelihoods and healthy diets in India.

While we did not investigate the direct effects of sickness in farming households, the subsequent lockdown policy was perceived by farmers to have affected production (through lack of labour, storage and inputs); sales (through drops in demand and lack of transport); prices and income (with reductions due to lack of demand); and diets (in terms of ability to access the most nutrient-dense foods). These core findings largely mirror the findings of previous research on food system disruptions reviewed above. A recent survey asking similar questions of farmers of various crops in India found similar impacts, with 10% of farmers not harvesting at all due to lockdown; vegetable farmers showing the worst impacts in terms of wastage; and reductions in nutrient-dense food consumption (Jaacks et al. 2020). A survey of vegetable value chains in Ethiopia similarly found increased farm losses alongside shortage of inputs and labour; reduced producer prices for vegetables, though retail prices so far unchanged; and both vegetable trade and consumption reduced (Tamru et al. 2020). These studies therefore concur broadly with what we saw in India. A study on retail prices in India found that these had increased and then stabilised in India on a national level, but that this varied by the particular type of vegetable (Pingali and Mittra 2020), an issue we were not able to address in this study. Indian newspapers have similarly reported issues with finding harvest labour, transport to market, reduced demand by buyers and retailers, and increased retail prices for vegetables in different states (Pothan et al. 2020). We look forward to further COVID-19 food system studies to compare our results further.

In addition to the core findings, sub-analyses of our data suggest that female farmers reported higher vulnerability in terms of both livelihood and diet. Women farmers claim to use more mitigation strategies to first secure their income, and then to secure their diet. The reasons women cite for changes in vegetable consumption show that they perceived themselves to be particularly affected by changes in food prices and affordability. So, the mitigation strategies available to them are not sufficient to protect their livelihoods and diets from income and price shocks.

Our findings suggest that farming households in general find it hard to mitigate the worst of these shocks in the short term, accepting lower prices to maintain sales, but still with interruptions to the flow of vegetables to consumers, thereby affecting diets more broadly in the country. Producing a variety of different vegetables appears to mitigate impact on sales (perhaps through diverse sales routes) but not on incomes (as prices are hit). The ability to consume one’s own produce is somewhat protective of diets when other routes to food access fail: due to the inability to sell their own produce, many households consumed more of their own vegetables and a proportion increased their consumption above previous levels. This route to dietary resilience is particular to the sample (horticultural households) but it does suggest that producing vegetables provides some resilience to dietary shocks for some households.

So far, the major responses of the Indian government to ensure livelihoods of farmers have been making loans more available and providing tax relief and direct farmer payments. In our work, smaller and larger farms see differences in the impacts of COVID-19, and therefore implement different coping strategies and require different types of support from the government in order to continue to function. Government response to ensure food security has been to double allocations under the Public Distribution System (PDS) and provide cash payments to out-of-work labourers (but not self-employed farmers). Female farmers in particular report accessing food aid according to our data, but it should be noted that the PDS system provides grain and pulses but not vegetables or other nutrient-dense foods that are seen to be badly hit in our data. Understanding farmers’ real-time coping strategies and limitations through studies such as this might help policy-makers to prepare strategies that take into account the lived experiences of farming households. The challenge is to carefully weigh the remaining direct epidemiological risks of the virus against the livelihood and dietary hazard to both consumers and farmers.

The impacts of COVID-19 and subsequent policy responses on both livelihoods and diets in horticultural households risk rolling back impressive economic and nutrition gains India has seen over the past decade. India’s Right to Food legislation commits the government to action in this area, and suggests that food systems, and particularly those making available the most nutrient-dense foods, must be considered in ongoing and future government responses.
